# Simple and efficient methods to generate split roots and grafted plants useful for long-distance signaling studies in *Medicago truncatula* and other small plants

**DOI:** 10.1186/1746-4811-8-38

**Published:** 2012-09-12

**Authors:** Tessema K Kassaw, Julia A Frugoli

**Affiliations:** 1 Department of Genetics and Biochemistry, Clemson University, 100 Jordan Hall, Clemson, SC, 29634, USA

**Keywords:** Split root, Inverted-Y, Grafting, Nodulation, Long-distance signaling, Systemic signaling, *Medicago truncatula*, RDN1

## Abstract

**Background:**

Long distance signaling is a common phenomenon in animal and plant development. In plants, lateral organs such as nodules and lateral roots are developmentally regulated by root-to-shoot and shoot-to-root long distance signaling. Grafting and split root experiments have been used in the past to study the systemic long distance effect of endogenous and environmental factors, however the potential of these techniques has not been fully realized because data replicates are often limited due to cumbersome and difficult approaches and many plant species with soft tissue are difficult to work with. Hence, developing simple and efficient methods for grafting and split root inoculation in these plants is of great importance.

**Results:**

We report a split root inoculation system for the small legume *M. truncatula* as well as robust and reliable techniques of inverted-Y grafting and reciprocal grafting. Although the split root technique has been historically used for a variety of experimental purposes, we made it simple, efficient and reproducible for *M. truncatula*. Using our split root experiments, we showed the systemic long distance suppression of nodulation on a second wild type root inoculated after a delay, as well as the lack of this suppression in mutants defective in autoregulation. We demonstrated inverted-Y grafting as a method to generate plants having two different root genotypes. We confirmed that our grafting method does not affect the normal growth and development of the inserted root; the composite plants maintained normal root morphology and anatomy. Shoot-to-root reciprocal grafts were efficiently made with a modification of this technique and, like standard grafts, demonstrate that the regulatory signal defective in *rdn1* mutants acts in the root.

**Conclusions:**

Our split root inoculation protocol shows marked improvement over existing methods in the number and quality of the roots produced. The dual functions of the inverted-Y grafting approach are demonstrated: it is a useful system to produce a plant having roots of two different genotypes and is also more efficient than published shoot-to-root reciprocal grafting techniques. Both techniques together allow dissection of long distance plant developmental regulation with very simple, efficient and reproducible approaches.

## Background

Signaling mechanisms are vital for all living organisms. Plants and animals use long-distance signals to coordinate and adjust their growth in response to endogenous and environmental cues. These signals transmit messages throughout the whole organism to achieve biological homeostasis. Plants use hormones, RNA, proteins, short peptides and lipids for long distance signaling in defense against pathogens [1], in response to numerous abiotic and biotic stresses [2] and in developmental processes such as flowering [3] branching [4] and nodulation [5,6]. Our lab uses *Medicago truncatula* to study long distance regulation; specifically the root-to-shoot and shoot-to-root signals that control nodule number and to understand the regulatory network involved in this process.

*M. truncatula* is an excellent model to study legume biology due to its small diploid genome (500 Mb), self-fertility, ease of transformation, short life cycle, high level of natural diversity and a wealth of genomic resources [7]. In addition, the bacterial and fungal symbionts of *M. truncatula* that lead to the fixation of nitrogen and the increased uptake of phosphorus are well-characterized [8,9]. In both rhizobial and arbuscular mycorrhizal symbioses, the establishment and maintenance of symbiosis requires expensive plant resources, specifically energy [10]. To balance this expense with other plant needs, legumes have a negative feedback inhibition system called autoregulation of nodulation (AON) [11,12]. Through AON, the early symbiotic events occurring in a root and leading to nodule organogenesis or to arbuscule formation systematically affect later symbiotic interactions through transportable signals. Efforts have been focused on locating time and space-specific root and shoot events including sensor, integrator and effector molecules. Grafting and split root inoculation systems can be very informative when combined with current molecular genetic tools to decipher the signaling. However, very little grafting and split root work has been done in model legumes, with historical work in less genetically tractable plants such as bean, pea, soybean, clover and vetch, some of which have stems much larger than those in model systems.

Establishment of symbiosis in one part of a legume root affects further symbiotic events in other parts of the root inoculated later, and this phenomenon was initially elucidated using split root experiments. In these split root systems, two roots in one plant are partitioned in time and space allowing prior inoculation of one root system (Root A) to systematically regulate nodulation from the separate inoculation of the second root (Root B). Around thirty years ago, it was reported that the suppression of nodule development in the Root B side of the split root system in soybean is associated with prior inoculation of the Root A side [5]. Five years later, Olsson et al. [13] reported the lack of systemic suppression of nodulation in supernodulating soybean mutants. Using a split root experiment, Tang, Robson and Dilworth [14] showed that iron is required for nodule initiation in lupine, emphasizing the direct and indirect impact of mineral nutrient deficiency on symbiosis. Application of either rhizobia or Nod factors to the Root A side of a split root system inhibits nodulation in the B root system, suggesting that Nod factors are enough to elicit the autoregulatory responses in vetch [15]. In clover a non-nodulating strain of *Rhizobium trifolii* inoculated on Root A was unable to inhibit infection by the wild type strain on Root B, suggesting a minimum requirement of Nod factor to initiate the plant inhibitory response [16]. In work by Laguerre, et al. [17] one root system was nodulated with a nitrogen-fixing bacterial partner while the other root system was nodulated with non-fixing partner, resulting in a plant that compensated for the local nitrogen limitation in the root with non-fixing bacteria. The same group had shown that in split root plants when one root is in a nitrogen-limited condition and the other receives nitrogen, both nitrogen fixation activity and net nitrogen uptake by the root system in the nitrogen-limited condition was higher in the *M. truncatula sunn-2* mutant versus wild type plants [18]. The authors suggested a secondary response of growth stimulation of pre-existing nodules in the wild type and *sunn-2* mutant. Autoregulation signals initiated by either nodulation or mycorrhization on Root A in alfalfa systemically influence both rhizobial and arbuscular mycorrhizal colonization of Root B in a split root system without preferential selection [19]. Also in alfalfa inoculated with mycorrhizae, isoflavonoid levels are systematically regulated in the uninoculated Root B upon prior inoculation of Root A, suggesting the involvement of isoflavonoids in the long distance autoregulation of arbuscular mycorrhizal symbiosis [20].

Developing a model of signal transduction by comparison across these experiments is difficult due not only to the use of many less tractable and less well developed molecular genetic systems with both determinate and indeterminate nodule development, but also to the use of a broad range of compartmentalization techniques to separate the split roots in various growth systems. For instance, PVC piping elbows have been used in soybean [5] and split root tubes have been used in soybean and vetch [5,13,15]. Split root plate assays were done using *Trifolium subterraneum* and *Lotus japonicus* by separating the roots with plastic dividers supported with 0.6% water agar [16] or by removing the center of the agar to create separate root environments [21]. The limitations of these techniques include inability to consistently control various factors known to affect nodule regulation, such as ethylene concentration in plate systems [22,23] and rhizobial cross contamination. Moreover, the effect on nodulation of root exposure to light [24], balancing the size of the root systems before treatment, the types of containers and the composition of the growth media including the amount of water and the concentration of various root exudates that affect nodulation were not consistently controlled. The above approaches were also targeted for very small laboratory scale applications, often with only 3–5 replicates (plants) per experiment.

In addition to the unintentionally introduced variables in the above experiments, many key factors in autoregulation remain unexplored in a single system/species. These factors include the time the AON signal takes to suppress nodulation and the stages of nodule initiation targeted by the AON signal. Except for the nitrogen experiments described above, the split root technique has not been used in *M. truncatula*. We were unable to find efficient examples of the use of the technique in model plants with growth parameters similar to *M. truncatula* (small stems approximately 0.1 cm in diameter). Hence improving the existing split root protocol to consistently generate large numbers of grafted plants was imperative for our AON investigations.

Another technique, reciprocal grafting, is also a valuable tool to study the remote and local interactions of various genotypes and systemic signals. For shoot-to-root reciprocal grafting in Arabidopsis, wedge grafting has been commonly employed, and adapted for other model plants with slight modifications [25-30]. Shoot-to-root reciprocal grafting allows researchers to examine the systemic signals and separate gene functions in above and below ground parts of the plant. The major limitations of the technique, especially in small plants, is the need for agar, parafilm, medical tubing or other physical support materials which interfere with inspection of the graft union and slow the healing of the union, sometimes influencing later plant development [31]. The success rate for *M. truncatula* reciprocal grafts is reported to be as low as 8% [32] and we have observed a rate of 50% depending on genotype in our previous work [27,29,30]; Lucinda Smith personal communication].

Despite the low success rate, reciprocal grafting is quite informative. Reciprocal grafting between a Zn hyperaccumulator, *Thlaspi caerulescens*, and a Zn nonaccumulator, *Thlaspi perfoliatum*, showed the relative importance of roots and shoots in Zn hyperaccumulation and hypertolerance [33]. The discovery of Flowering Locus T (FT) protein as a long distance signal moving from the leaf to the apex through phloem to induce flowering in Arabidopsis was done with grafting [3]. Grafting analysis provided evidence that the shoot genotype controls the supernodulating phenotype in the autoregulation defective mutants *har1* and *klv* in *Lotus japonicus*[34,35], *sym29* in *Pisium sativum*[26], *nark* in *Glycine max*[36], *sunn* and *lss* in *M. truncatula*[27,29]. For example, grafting *sunn* and *lss* scions on wild type (A17) rootstock produced a hypernodulation phenotype whereas the reciprocal grafting of A17 scion on either *sunn* or *lss* rootstock gave wild type nodulation phenotype [27,29]. Grafting also revealed the action of the *rootdeterminednodulation 1* mutant *rdn1* in which, unlike the examples above, the root genotype controls the hypernodulation phenotype [30]. In cases of root-determined hypernodulation, the cause could be a defect in either the synthesis or transmission of the root derived factor or the transport and/or perception of the shoot derived descending inhibitory signal. Distinguishing between these possibilities requires a plant with roots of two different genotypes. Working in pea, researchers used approach grafting between wild type pea and lines with mutations affecting AON to reveal that early nodulation events prior to root hair curling cannot induce the AON signal, demonstrating that AON starts after root hair curling but before visible cortical and pericycle cell division [37]. However approach grafting, in which two complete plants are joined in the stem region, adds the complication of having two shoots of different genotypes that may vary in their vascular connections to the roots and their production of the unknown shoot derived inhibitory signal compared to a single shoot, making the findings from these experiments difficult to interpret definitively. Therefore we developed an inverted-Y grafting technique to provide an extra dimension to the split root experiment by partitioning the two roots of the same plant not only in time and space but also in genotype. This experimental approach allows for the dissection of function of the gene products involved in the regulatory circuit without the complications created by approach grafting.

This methodology report describes these highly efficient split root and inverted-Y grafting protocols in *M. truncatula*. Our techniques provide simple ways of generating many root systems to dissect long distance signaling. These can be either split root experiments where the effect of a treatment on one root is detected on the second root or inverted-Y graft experiments where plants with two different root genotypes are used to study the root-to-shoot and shoot-to-root signals and individual gene actions. We also report a modification of the inverted-Y technique that allows rapid generation of a large number of reciprocally grafted plants with a single shoot and single root.

## Results

### A split root technique demonstrates systemic AON

**Figure 1:**
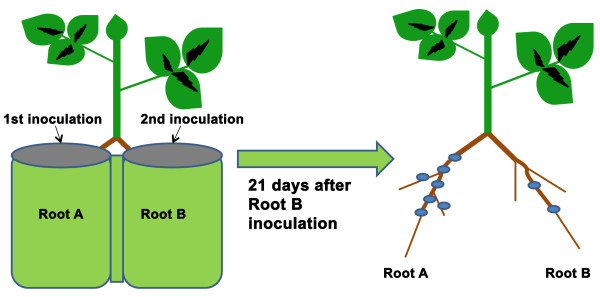
**Generalized diagram of split root inoculation in *M. truncatula. ***A plant with two main roots is created via our protocols and each root is placed in Perlite in a separate pot. One root is inoculated first (Root A) and the second root (Root B) is inoculated at a later time point. The plants continue to grow for 21 days after the second inoculation, at which point they are removed from the Perlite system and nodules on each root are counted.

**Figure 2:**
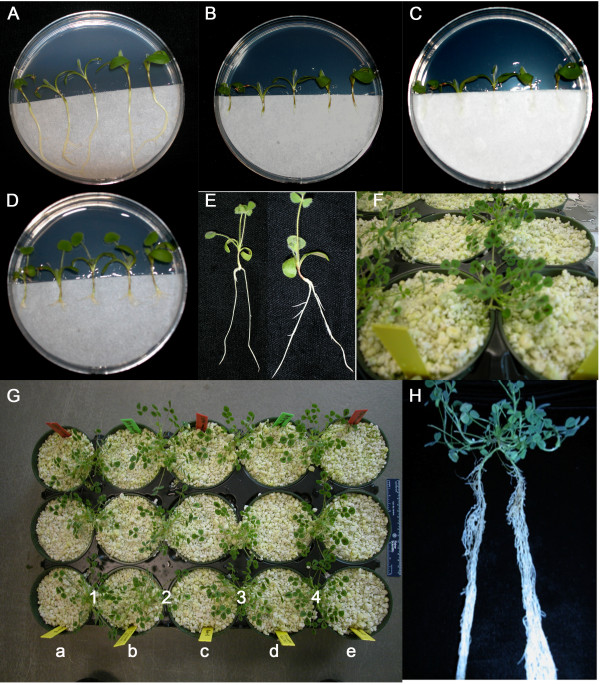
**Generating split roots in *M. truncatula. ***(**A**) Plants on Petri dishes before cutting roots, (**B**) after cutting roots and (**C**) after placing filter paper over the roots. (**D**) Lateral root initiation 5 days after cutting the root. (**E**) Example of 2 plants trimmed to two balanced root systems ready for transfer to Perlite. (**F**) Plants with two root systems, one growing in each pot (**G**) Experimental design of pots in a standard greenhouse tray that maximizes plants per unit space. Each row is a replicate, and each column contains the following (a) Root A of plant 1 (b) Root B of plant 1 and Root B of plant 2 (c) Root A of plant 2 and Root A of plant 3 (d) Root B of plant 3 and Root B of plant 4 (e) Root A of plant 4 (**H**) A plant 21 days after Root B inoculation ready for counting.

**Figure 3:**
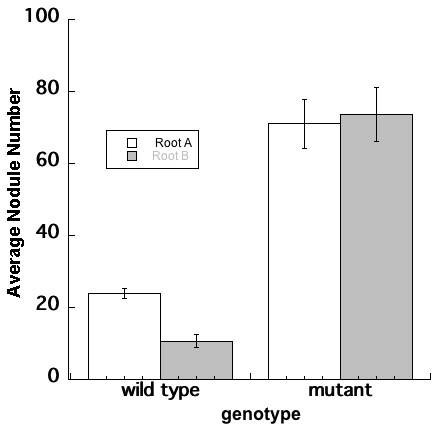
**Split root plants demonstrate that the suppression of nodulation is lost in an AON mutant.** Number of nodules per root on the root inoculated first (Root A) and the root inoculated 4 days later (Root B) counted 21 days after second inoculation. The wild type is ecotype *A17* and the mutant *rdn1-2*. Data represent means (N = 6 for A17, N = 23 for *rdn1-2*). Error bars are standard error of the mean. Letters (a, b and c) indicate significant differences between Root A and B, and between the two genotypes (p < 0.05).

Split-root experiments are valuable for the investigation of the autoregulation of nodulation, i.e. systemic suppression of subsequent root colonization by rhizobia through signaling from an already colonized part of the root system. We developed a split root inoculation protocol for *M. truncatula* by modifying an existing *Agrobacterium* mediated hairy root transformation method for *M. truncatula*[38] diagrammed in Figure 1 and detailed in Figure 2. Briefly, the transformation protocol was followed to the point of removing the root system (Figure 2A & B) and placing the plants on HMF media sandwiched between two half round Whatman filter papers (Figure 2C), but no *Agrobacterium* was applied. Lateral roots were allowed to grow out from the cut (Figure 2D). Two lateral roots of the same size were selected (Figure 2E), while the rest of the roots were removed and the two remaining roots grown in separate root environments by placing them in side-by-side plastic pots in a Perlite system with free drainage (Figure 2F). By combining roots from alternate plants in a single pot (Figure 2G), four split root systems could be accommodated in five pots. One side of the split root system (Root A) was inoculated with *Sinorhizobium medicae* strain ABS7 [39] and the other side of the split root system (Root B) was inoculated four days after the first root inoculation with the same rhizobium strain at the same concentration. In agreement with experiments in soybean [5], wild type (A17) roots inoculated second (Root B) had significantly fewer nodules than the root inoculated first (Root A) (Figure 3). As might be expected, in *rdn1* mutants known to make too many nodules [30], inoculation of the first root had no effect on nodule number on the second root, confirming a defect in AON (Figure 3).

### Inverted-Y graft plants respond normally to the transmissible signal

The main objective of inverted-Y grafting is to obtain a graft of one shoot to two different rootstock genotypes in the same plant in order to understand the role and timing of action in long distance regulation of a mutant gene. Since graft union development is a delicate process that involves cell-to-cell communication, there are many factors that lead to poor healing and connection of the graft, such as differences in morphology between the stock and the scion and the water content of the media [31]. Yin et al. used an oblique medium surface to alleviate these problems [31]. Based on this idea, we modified an inverted-Y grafting approach similar to that recently used by Magori [28] in *Lotus japonicas* for *M. truncatula* and noted its applicability for other model plants.

**Figure 4:**
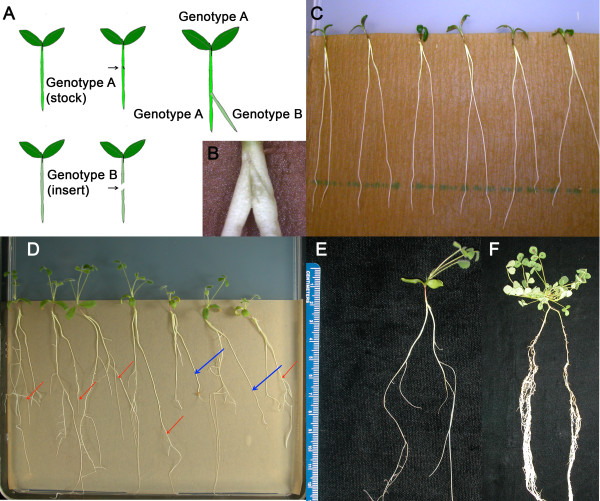
**Creating an inverted-Y graft between two different rootstock genotypes.** (**A**) Schematic representation of inverted-Y grafting. Genotype A is the main stock which contributes both the shoot genotype and one of the rootstocks and genotype B is acting as the insert contributing only one of the rootstocks. (**B**) Close up of graft immediately after insertion. (**D**) Four day old plants immediately after grafting on BNM media covered with brown seed germination paper. (D and E) Successful grafts immediately before transferring to a Perlite system. Red arrows in C indicate the new root growth in the successful grafts while blue arrows indicate failed grafts that lack new growth. (**F**) Representative plant 21 days after the second root inoculation; washed and ready for nodule counting.

We grew *M. truncatula* wild type plants on buffered nodulation media (BNM-see Materials & Methods) for four to five days and performed wild type to wild type inverted-Y grafts to demonstrate the protocol. Two approximately equal sized stock and insert roots were selected for grafting. Plants with rootstocks originating with two different plants were obtained by diagonally cutting completely through the insert root (Root B) at root-shoot junction, while making small incision on the same spot of the stock root (Root A) with same angle as the insert and grafting the insert upwards into the silt of the stock so that the cut surface of the insert faces the path of the stock (Figure 4A). Unlike other protocols in the literature [4,37,40], this technique does not require parafilm, medical tubing or other support materials. Instead the two roots support each other until the graft is healed (Figure 4B). Our experience in developing the *M. truncatula* inverted-Y grafting approach agrees with the comments of Yin, et al. on water and morphology differences [31]; we too have incidentally observed that the healing process appears to be negatively affected by the water content of the media. We overcame this by using thick pre-wetted brown seed germination paper on BNM media to create a slightly dryer environment than agar alone and facilitated the healing process by incubating the plates horizontally for five days (Figure 4B). Successful grafting was determined by new root growth 2–3 weeks after grafting (Figure 4C). Plants with healed roots of equal size (Figure 4D) were then transferred to Perlite and grown/inoculated as in the split root protocol (Figure 2F). Because the only way to visually distinguish roots of different genotypes on plates is labeling, we cannot emphasize enough the importance of attention to detail in the labeling of the plates and pots.

**Figure 5:**
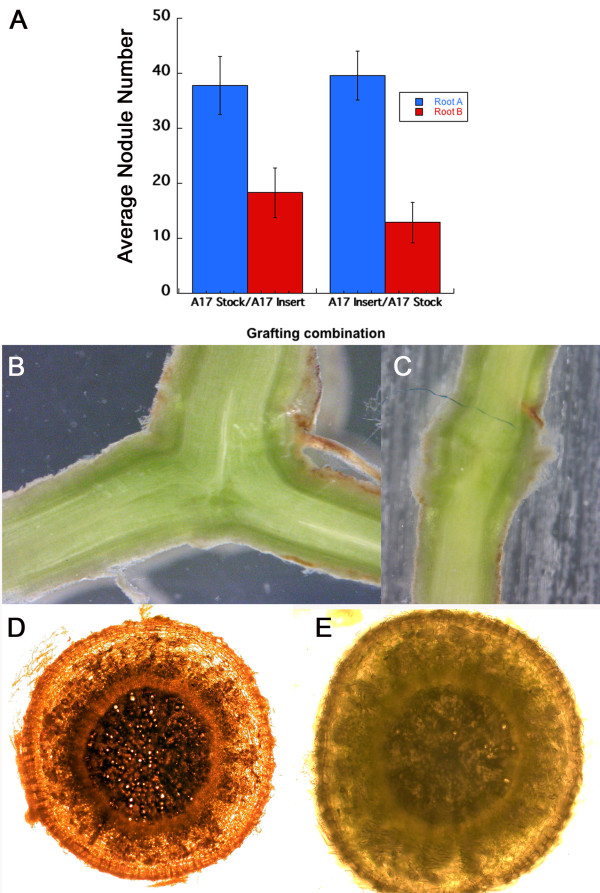
**Nodule number on inverted-Y grafted plants.** (**A**) Wild type (A17) self grafts show systemic inhibition of nodulation on the second root. The X axis label indicates whether the first root inoculated (Root A) was the stock root or the inserted root. Root B was inoculated 4 days after Root A and data was collected 21 days after Root B inoculation. The Y axis is average nodule number per root; error bars indicate standard error of the mean. Both comparisons are significant by pair-wise t-test with P < 0.05. N = 6 for the first set of bars and 8 for the second. (**B**) Longitudinal section of vasculature at Y graft junction (**C**) Reciprocal graft longitudinal section. (**D** &**E**) Cross sections of stock root and grafted root respectively of plant in (**B**).

The wild type to wild type grafts resulted in large root systems and the plants nodulated normally upon rhizobial inoculation (Figure 4E). Consistent with our split root inoculation experiment, the initial inoculation of Root A systemically suppressed nodule formation on the Root B demonstrating the grafted roots behaved as a single system. In order to confirm that there was no effect of the healing process on the graft transmissible signal, we also used the grafted root and the main stock root interchangeably as “Root A” and inoculated with rhizobia first. As indicated by comparing the two combinations in Figure 5A, inoculating either root first did not affect the systemic suppression of nodulation in the respective second root. This suggests that the grafted root is functionally and morphologically identical to the main root and the transmissible signal is not affected by the procedure. As further confirmation of vascular integrity, we performed microscopic analysis of cross sections of the Y graft junctions and noted normal vascular connections (Figure 5B, D, E).

### The inverted-Y grafting approach can also be used for shoot to root reciprocal grafting

**Figure 6:**
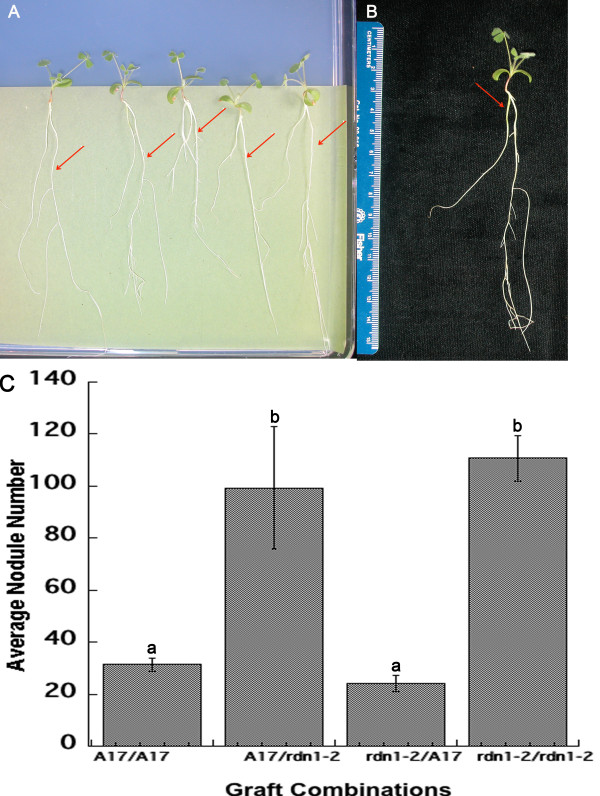
**Shoot to root reciprocal grafting.** (A & B) Successfully grafted plants on BNM plates immediately before planting on Perlite. (**A**) The actively growing roots (indicated by red arrows) are the insert roots; the stock roots have ceased growth. (**B**) Yellow arrow marks root to be cut before transfer to Perlite. (**C**) Average nodule number on reciprocally grafted *M. truncatula* plants. Error bars indicate standard error of the mean. For the combinations noted as shoot/root, N = 27 for A17/A17, 4 for A17/*rdn1-2*, 4 for *rdn1-2*/A17 and 20 for *rdn1-2/rdn1-2*. Letters designate significant difference by Tukey-Kramer test (p < 0.05).

As noted, there is a long history of reciprocal grafting, at least in larger species, to demonstrate transmissible signals. However, in *Medicago truncatula* the root/shoot junction and the roots themselves are soft and have a narrow diameter making them difficult to handle during standard grafting procedures. By using the above inverted-y grafting approach with slight modifications (cutting deeper into the hypocotyl of the main root and then removing the original root after the graft heals), we improved the success rate of reciprocal grafts from 50% in our previous work [27,29,30] and 8% in that of Lohar [32] to 66% (Table 1). This deeper cut prevents the stock root from growing and favors the healing of the vasculature in the new root. Successful grafts, indicated by fresh root growth on the inserted root (Figure 6A), were selected while the main rootstocks, which usually did not show further development due to the deep cutting, were removed by excision with a razor blade before planting (Figure 6B).

**Table 1:** Success rate of shoot to root reciprocal grafting using a modification of the inverted-Y technique

**Graft combination**	**Grafts initiated**	**Successful grafts**	**Success rate (%)**
Wild type/Wild type	24	18	75
Mutant-1/Mutant-1	21	11	52.4
Mutant-2/Mutant-2	23	14	60.9
Mutant-1/Wild type	12	10	83.3
Mutant-1/Mutant-2	23	14	60.9
Mutant-2/Mutant-1	24	15	62.5
**Mean**/-**SE**	**21.2**/-**1.9**	**13.7**/-**1.2**	**65.8**/-**4.6**

The description of the graft combination refers to Wild type:ecotype Jemalong A17, mutant-1: the shoot controlled hypernodulator *sunn-4*[41] and mutant-2: the root controlled hypernodulator *rdn1-2*[30].

Figure 6C shows a comparison of nodule number in reciprocal and self-grafted plants between the wild type (A17) and the autoregulation defective mutant *rdn1-2* 21 days post inoculation. Hypernodulation resulted when a wild type shoot was grafted onto an *rdn1-2* root. In contrast, wild type nodulation resulted when an *rdn1-2* shoot was grafted onto a wild type root, in agreement with the conclusion of our previous grafting studies that nodule number in *rdn1-2* is determined by the root genotype [30]. However, the use of our new technique added statistical power to our observations by allowing us to increase the number of plants used in a given experiment up to 20. Indeed our new approach also provided healthy grafts morphologically indistinguishable from each other and intact plants except for the number of nodules (Figure 5C, Additional file 1: Figure S1).

## Discussion

This study presents two improved and reliable techniques of dissecting long distance autoregulatory mechanisms of nodule regulation in *M. truncatula*; (1) a split root experiment to assay AON inhibition that can be adapted to test long distance signaling in any combination of root environments and plants that can be grown on plates and moved to Perlite and (2) an inverted-Y graft experiment that in addition to the above benefits, can be used to differentiate signaling originating in the root from downstream roots responses to shoot signals and that can also be used to generate reciprocal grafts.

The ease of the described split root protocol increases the number of split root plants most investigators can generate for a particular experiment by growing the split root seedlings on HMF media until they are ready for planting on soil system. In addition, the planting of the separate root systems does not require PVC piping or special split root tubes. Instead, ten days after the cutting of the primary root, the regenerated lateral roots are long enough to plant directly in separate pots filled with washed Perlite. The initiation of lateral root development on HMF media sandwiched between two half round Whatman filter papers provided the option to select two equivalent roots and trim the rest for the split root assay (Figure 2). The choice of the Perlite growth system for the nodulation portion of the assay allows ethylene to dissipate, eliminating a significant variable in root response. We demonstrated the inhibitory effect of the autoregulation signal in the delayed inoculated wild type root and showed that the *rdn1-2* mutant is defective in AON (Figure 3). We are now using the techniques to define precisely when the AON circuit initiates, how long it lasts, and whether there is any second message after the initial signal.

Grafting is a valuable tool for dissection of long distance plant communication between roots and shoots. The advantage of the grafting methods presented here is the elimination of the need to hold the grafts together with extra materials, making the method applicable for plants with very small stem diameters. Instead of two shoots from approach grafting [37], an inverted-Y graft provides a single shoot that perceives the root derived factor and transduces the shoot derived inhibitory signal. Because our grafting technique does not need materials for physical support (Figure 4), it also requires less manual dexterity by the experimenter and should be suitable for growing plants out on soil, plates and in aeroponic chambers after grafting. The success rate is high for a complex technique; in our hands it is so high that we routinely make the less complex reciprocal grafts by the modification described to the inverted-Y experiment rather than the technique used in our previous work [27,29,30]. Using the inverted-Y method, we demonstrated that the shoot derived inhibitory signal initiated by inoculating the one of the roots (Root A) first suppresses nodulation in the delayed inoculated second root (Root B). We also confirmed the success of the graft union and the intact nature of cell-to-cell communication in the plants by showing that the results were not dependent on whether the grafted root or the original root was inoculated first (Figure 5). Currently, using the inverted-Y technique we are investigating the role of *RDN1* in long distance nodule regulation. Taken together, these data suggest that the inverted-Y graft will be invaluable in identifying the different components of the AON circuit by grafting different combinations of wild type and the autoregulation defective mutants together.

The modification of the inverted-Y grafting technique we developed for shoot-to-root reciprocal grafting is simple and highly efficient, with a 66% success rate in plants carrying two different mutations in genes of the AON pathway (Table 1). It does not require external support as the stock root will act as a support until the graft heals. Most importantly, the grafting method does not affect the normal development of the plant since healthy and completely healed grafts are attained rapidly and the morphology and nodulation of the grafted seedlings are the same as in the intact wild type and mutant plants. Using this approach, we demonstrated that *rdn1* mutants are defective in sending the root derived signal initiated by inoculation or perceiving the shoot derived inhibitory signal confirming the *rdn1* mutation is appropriately named *rootdeterminednodulator* (Figure 6 and [30]).

## Conclusion

The described methods are useful for the dissection of long distance signaling in plants. Our split root inoculation protocol showed marked improvement over existing methods by eliminating complex apparatuses and allowing selection of root systems balanced for size. Elimination of external supports coupled with defined growth conditions allows even dexterity challenged experimenters to create experimental plants with split root systems. The inverted-Y graft approach has the dual advantage of being more efficient than published shoot-to-root reciprocal grafting techniques and is also a useful system to produce a plant having two different rootstock genotypes. Both techniques together allow dissection of long distance plant developmental regulation using very simple, efficient and reproducible techniques. We suggest the combination of autoregulation defective mutants, grafting, and inoculation of split root systems will complement other molecular genetic and biochemical approaches to unravel the signal transduction involved in AON. We propose the techniques should be broadly applicable to other plants, including those of small size such as Arabidopsis.

## Materials and methods

### Plant materials and growth conditions

Seeds of *Medicago truncatula* cv Jemalong ecotype A17 and the root determined nodulator *rdn1-2*[30] were utilized for this experiment. Seeds were acid scarified and imbibed as described in Schnabel et al. [30]. Seeds were vernalized in dark at 4°C for 2 days on Harrison Modified Farhaeus (HMF) media [42] covered with two half round filter papers (Whatman, catalog # 1001090). The seeds were then germinated in the dark at room temperature for 1 day and used for the following techniques.

### Split root technique

Using sterile technique under a positive flow hood (for all work described in this report done before transfer to Perlite), one day old seedlings were placed five seedlings per plate on 9 cm Petri dishes containing HMF media covered with sterilized half round filter papers (Whatman) and allowed to grow vertically for five days in a growth chamber (25°C and 16 hr photoperiod). Lateral root initiation was stimulated by removing the roots of these five day old seedlings at the root-shoot junction and transferring them to new HMF plates sandwiched between two half round Whatman filter papers, important to keep the roots moist and in the dark. We used a sterile razor blade to cut the root under aseptic conditions. After growing vertically in the growth chamber for an additional five days, the top filter paper was removed. Approximately one week later, lateral roots of sufficient length had formed and plants having two adventitious roots of approximately equal size were chosen for planting in split-root pots filled with washed Perlite (Figure 2E). Each of these two lateral roots (Root A and Root B in Figure 1) was separately planted to an individual pot, and the remaining lateral roots were cut off. To establish the plants in the Perlite system, the plants were watered for 5 days with a 100-fold dilution of water-soluble 20:10:20 Peat-Lite Special fertilizer (Scotts). Fertilization was then withdrawn and the plants were hydrated with water alone an additional five days in order to induce nitrogen deficiency required for nodulation. The plants were then used for split-root inoculation experiments with bacteria. The first root (Root A) was inoculated via flood inoculation (see inoculation below) and second root (Root B) was inoculated in the same manner four days later. Nodules on each root were counted 21 days after the Root B inoculation.

### Inverted-Y grafting

One day old seedlings were grown on sterile Nunc Bio-Assay dishes (245 mm x 245 mm x 25 mm) each containing 250 ml of BNM [43] covered with 16.5 cm x 22.9 cm brown seed germination paper (Anchor Paper 76 # heavy weight brown seed germination paper) and sealed with Curasilk™ Hypoallergenic cloth tape (Kendall). We found this particular weight of paper to be important for good results. The seedlings were grown vertically for 5 days in the growth chamber at 25°C with a 16 hr photoperiod. Relatively equal roots from the respective stock and insert genotypes were selected for grafting. Using a sterile razor blade under aseptic conditions in a positive flow hood, the insert root was diagonally sliced at the hypocotyl, while making a small incision (slit) about half way through the hypocotyl of the stock root with same angle as the insert. In our experience, cutting deeper than halfway into the stem prevents the stock root from growing, presumably because the vasculature is damaged, and the delay in growth affects the healing of the graft favoring the new root over the stock root. The graft was inserted upwards into the slit of the stock so that the cut surface of the insert faced the path of the stock. The plates were sealed again with Curasilk™ Hypoallergenic cloth tape (Kendall). Five days after grafting, the plates were turned vertically and the grafted plants allowed to grow for more than ten days under the same growth chamber conditions. Occasionally (at most twice in an experiment) any extra lateral roots coming out from the stock were trimmed to stimulate the growth of the inserted root. After at least 10 days, the well-grafted seedlings (determined by new growth on both roots) were transferred into the Perlite system and grown and evaluated as in the split root experiment described above.

### Inverted-Y grafting for reciprocal grafts

For shoot to root reciprocal grafting, the inverted-Y grafting was performed as mentioned above except the incision for inserting the graft was cut diagonally 75–80 percent into the hypocotyl of the stock, with same angle as the insert. As mentioned in the methodology for the inverted-Y graft, this prevents the stock root from growing and favors the graft to preferentially heal the new root. Successful grafts, indicated by fresh root growth on the inserted root, were selected and the main rootstocks, which usually did not show further development due to the deep cutting, were removed by excision with a razor blade before planting.

### Inoculation & sectioning

All plants were inoculated with *Sinorhizobium medicae* strain ABS7, carrying a LacZ reporter gene on a plasmid with tetracycline resistance [39]. The rhizobia were grown in liquid TY media containing 15 μg/ml tetracycline at 30°C on a rotary shaker at 250 rpm for approximately 48 hrs. Before inoculation, the rhizobia were diluted to an OD 600 nm of 0.2 with water and 6 ml of the bacterial solution was applied to each root compartment at root collar region (flood inoculation). Nodulation was observed and nodules were counted 21 days after the second root inoculation for both the split root and inverted-Y graft experiment and 21 days after the only inoculation for the reciprocal grafting. Nodules were counted using an Olympus SZX12 Dissecting Stereo Microscope after gentle washing of the Perlite away from the roots. Hand sections of roots were made with a sharp razor blade while holding the root down with forceps.

### Photography and data analysis

Roots were photographed with the same Olympus SZX12 Dissecting Stereo Microscope using an Olympus DP11 Digital Camera System or a Zeiss Axiostar plus with a Nikon coolpix5000 digital camera for the cross sections in Figure 5C & D. Larger pictures were also obtained with the Nikon coolpix5000 camera. For split root inoculation and inverted-Y graft data paired t-tests were used to identify statistical significance differences between root systems using p < 0.05 as the significance level. The Tukey-Kramer test was used for shoot to root reciprocal grafted data (p < 0.05).

## Competing interests

The authors declare that they have no competing interests.

## Authors’ contributions

TK participated in the design of the experiments, carried out the experiments, and drafted the manuscript. JF also participated in the design and coordination of the experiments and helped write the manuscript. Both authors read and approved the final manuscript.

## Supplementary Material

Additional file 1 **Figure S1.** Washed reciprocally grafted plants harvested from the soil ready for nodule counting showing large, healthy root systems. Click here for file

## References

[B1] Heil M Ton J Long-distance signalling in plant defence Trends Plant Sci 2008 13 264 272 10.1016/j.tplants.2008.03.005 18487073

[B2] Pant BD Buhtz A Kehr J Scheible WR MicroRNA399 is a long-distance signal for the regulation of plant phosphate homeostasis Plant J 2008 53 731 738 10.1111/j.1365-313X.2007.03363.x 17988220 PMC2268993

[B3] Corbesier L Vincent C Jang S Fornara F Fan Q Searle I Giakountis A Farrona S Gissot L Turnbull C Coupland G FT protein movement contributes to long-distance signaling in floral induction of Arabidopsis Science 2007 316 1030 1033 10.1126/science.1141752 17446353

[B4] Foo E Turnbull CG Beveridge CA Long-distance signaling and the control of branching in the rms1 mutant of pea Plant Physiol 2001 126 203 209 10.1104/pp.126.1.203 11351083 PMC102294

[B5] Kosslak RM Bohlool BB Suppression of nodule development of one side of a split-root system of soybeans caused by prior inoculation of the other side Plant Physiol 1984 75 125 130 10.1104/pp.75.1.125 16663555 PMC1066847

[B6] van Noorden GE Ross JJ Reid JB Rolfe BG Mathesius U Defective long-distance auxin transport regulation in the Medicago truncatula super numeric nodules mutant Plant Physiol 2006 140 1494 1506 10.1104/pp.105.075879 16489131 PMC1435797

[B7] Cook DR Medicago truncatula–a model in the making! Curr Opin Plant Biol 1999 2 301 304 10.1016/S1369-5266(99)80053-3 10459004

[B8] Galibert F Finan TM Long SR Puhler A Abola P Ampe F Barloy-Hubler F Barnett MJ Becker A Boistard P Bothe G Boutry M Bowser L Buhrmester J Cadieu E Capela D Chain P Cowie A Davis RW Dreano S Federspiel NA Fisher RF Gloux S Godrie T Goffeau A Golding B Gouzy J Gurjal M Hernandez-Lucas I Hong A The composite genome of the legume symbiont Sinorhizobium meliloti Science 2001 293 668 672 10.1126/science.1060966 11474104

[B9] Martin F Gianinazzi-Pearson V Hijri M Lammers P Requena N Sanders IR Shachar-Hill Y Shapiro H Tuskan GA Young JP The long hard road to a completed Glomus intraradices genome New Phytol 2008 180 747 750 10.1111/j.1469-8137.2008.02671.x 19138232

[B10] Crawford NM Kahn ML Leustek T Long SR Buchanan BB, Gruissem W, Jones RL Nitrogen and sulfur Biochemistry and Molecular Biology of Plants 2000 Rockville: American Association of Plant Physiologists 786 846

[B11] Pierce M Bauer WD A rapid regulatory response governing nodulation in soybean Plant Physiol 1983 73 286 290 10.1104/pp.73.2.286 16663209 PMC1066454

[B12] Searle IR Men AE Laniya TS Buzas DM Iturbe-Ormaetxe I Carroll BJ Gresshoff PM Long-distance signaling in nodulation directed by a CLAVATA1-like receptor kinase Science 2003 299 109 112 10.1126/science.1077937 12411574

[B13] Olsson JE Nakao P Bohlool BB Gresshoff PM Lack of systemic suppression of nodulation in split root systems of supernodulating soybean (Glycine max [L.] merr.) mutants Plant Physiol 1989 90 1347 1352 10.1104/pp.90.4.1347 16666934 PMC1061894

[B14] Tang C Robson AD Dilworth MJ A split-root experiment shows that iron is required for nodule initiation in Lupinus angustifolius L New Phytol 1990 115 61 67 10.1111/j.1469-8137.1990.tb00922.x 33874148

[B15] van Brussel AA Tak T Boot KJ Kijne JW Autoregulation of root nodule formation: Signals of both symbiotic partners studied in a split-root system of Vicia sativa subsp. nigra Mol Plant Microbe Interact 2002 15 341 349 10.1094/MPMI.2002.15.4.341 12026172

[B16] Sargent L Huang SZ Rolfe BG Djordjevic MA Split-root assays using Trifolium subterraneum show that rhizobium infection induces a systemic response that can inhibit nodulation of another invasive rhizobium strain Appl Environ Microbiol 1987 53 1611 1619 16347390 10.1128/aem.53.7.1611-1619.1987 PMC203919

[B17] Laguerre G Heulin-Gotty K Brunel B Klonowska A Le Quere A Tillard P Prin Y Cleyet-Marel JC Lepetit M Local and systemic N signaling are involved in Medicago truncatula preference for the most efficient Sinorhizobium symbiotic partners New Phytol 2012 195 437 449 10.1111/j.1469-8137.2012.04159.x 22548481

[B18] Jeudy C Ruffel S Freixes S Tillard P Santoni AL Morel S Journet EP Duc G Gojon A Lepetit M Salon C Adaptation of Medicago truncatula to nitrogen limitation is modulated via local and systemic nodule developmental responses New Phytol 2010 185 817 828 10.1111/j.1469-8137.2009.03103.x 20015066

[B19] Catford JG Staehelin C Lerat S Piche Y Vierheilig H Suppression of arbuscular mycorrhizal colonization and nodulation in split-root systems of alfalfa after pre-inoculation and treatment with nod factors J Exp Bot 2003 54 1481 1487 10.1093/jxb/erg156 12709494

[B20] Catford J Staehelin C Larose G Piché Y Vierheilig H Systemically suppressed isoflavonoids and their stimulating effects on nodulation and mycorrhization in alfalfa split-root systems Plant Soil 2006 285 257 266 10.1007/s11104-006-9012-8

[B21] Suzuki A Hara H Kinoue T Abe M Uchiumi T Kucho K Higashi S Hirsch AM Arima S Split-root study of autoregulation of nodulation in the model legume Lotus japonicus J Plant Res 2008 121 245 249 10.1007/s10265-007-0145-5 18202823

[B22] Penmetsa RV Cook DR A legume ethylene-insensitive mutant hyperinfected by its rhizobial symbiont Science 1997 275 527 530 10.1126/science.275.5299.527 8999796

[B23] Tamimi SM Timko MP Effects of ethylene and inhibitors of ethylene synthesis and action on nodulation in common bean (Phaseolus vulgaris L.) Plant Soil 2003 257 125 131

[B24] Webb D Effects of light on root growth, nodulation, and apogeotropism of Zamia pumila L. seedlings in sterile culture Am J Bot 1982 69 298 305 10.2307/2443017

[B25] Turnbull CG Booker JP Leyser HM Micrografting techniques for testing long-distance signalling in Arabidopsis Plant J 2002 32 255 262 10.1046/j.1365-313X.2002.01419.x 12383090

[B26] Nishimura R Hayashi M Wu GJ Kouchi H Imaizumi-Anraku H Murakami Y Kawasaki S Akao S Ohmori M Nagasawa M Harada K Kawaguchi M HAR1 mediates systemic regulation of symbiotic organ development Nature 2002 420 426 429 10.1038/nature01231 12442172

[B27] Penmetsa RV Frugoli J Smith L Long SR Cook D Genetic evidence for dual pathway control of nodule number in Medicago truncatula Plant Physiol 2003 131 998 1008 10.1104/pp.015677 12644652 PMC166865

[B28] Magori S Kawaguchi M Long-distance control of nodulation: Molecules and models Mol Cells 2009 27 129 134 10.1007/s10059-009-0016-0 19277493

[B29] Schnabel E Mukherjee A Smith L Kassaw T Long S Frugoli J The lss supernodulation mutant of Medicago truncatula reduces expression of the SUNN gene Plant Physiol 2010 154 1390 1402 10.1104/pp.110.164889 20861425 PMC2971615

[B30] Schnabel EL Kassaw TK Smith LS Marsh JF Oldroyd GE Long SR Frugoli JA The ROOT DETERMINED NODULATION1 gene regulates nodule number in roots of Medicago truncatula and defines a highly conserved, uncharacterized plant gene family Plant Physiol 2011 157 328 340 10.1104/pp.111.178756 21742814 PMC3165882

[B31] Yin H Yan B Sun J Jia P Zhang Z Yan X Chai J Ren Z Zheng G Liu H Graft-union development: A delicate process that involves cell–cell communication between scion and stock for local auxin accumulation J Exp Bot 2012 10.1093/jxb/ers109 PMC3398452 22511803

[B32] Lohar DP VandenBosch KA Grafting between model legumes demonstrates roles for roots and shoots in determining nodule type and host/rhizobia specificity J Exp Bot 2005 56 1643 1650 10.1093/jxb/eri160 15824071

[B33] Guimarães MDA Gustin JL Salt DE Reciprocal grafting separates the roles of the root and shoot in zinc hyperaccumulation in Thlaspi caerulescens New Phytol 2009 184 323 329 10.1111/j.1469-8137.2009.02969.x 19656301 PMC2784906

[B34] Krusell L Madsen LH Sato S Aubert G Genua A Szczyglowski K Duc G Kaneko T Tabata S De Bruijn FJ Pajuelo E Sandal N Stougaard J Shoot control of root development and nodulation is mediated by a receptor-like kinase Nature 2002 420 422 426 10.1038/nature01207 12442170

[B35] Miyazawa H Oka-Kira E Sato N Takahashi H Wu GJ Sato S Hayashi M Betsuyaku S Nakazono M Tabata S Harada K Sawa S Fukuda H Kawaguchi M The receptor-like kinase KLAVIER mediates systemic regulation of nodulation and non-symbiotic shoot development in Lotus japonicus Development 2010 137 4317 4325 10.1242/dev.058891 21098572

[B36] Delves AC Mathews A Day DA Carter AB Carroll BJ Gresshoff PM Regulation of the soybean rhizobium nodule symbiosis by shoot and root factors Plant Physiol 1986 82 588 590 10.1104/pp.82.2.588 16665072 PMC1056163

[B37] Li D Kinkema M Gresshoff PM Autoregulatio of nodulation (AON) in Pisum sativum (pea) involves signalling events associated with both nodule primordia development and nitrogen fixation J Plant Physiol 2009 166 955 967 10.1016/j.jplph.2009.03.004 19403196

[B38] Limpens E Ramos J Franken C Raz V Compaan B Franssen H Bisseling T Geurts R RNA interference in Agrobacterium rhizogenes-transformed roots of Arabidopsis and Medicago truncatula J Exp Bot 2004 55 983 992 10.1093/jxb/erh122 15073217

[B39] Bekki A Trichant JC Rigaud J Nitrogen fixation (C2H2 reduction) by Medicago nodules and bacteroids under sodium chloride stress Physiol Plant 1987 71 61 67 10.1111/j.1399-3054.1987.tb04617.x

[B40] Li B Wang Y Zhang Z Wang B Eneji AE Duan L Li Z Tian X Cotton shoot plays a major role in mediating senescence induced by potassium deficiency J Plant Physiol 2012 169 327 335 10.1016/j.jplph.2011.10.009 22154601

[B41] Schnabel E Journet EP de Carvalho-Niebel F Duc G Frugoli J The Medicago truncatula SUNN gene encodes a CLV1-like leucine-rich repeat receptor kinase that regulates nodule number and root length Plant Mol Biol 2005 58 809 822 10.1007/s11103-005-8102-y 16240175

[B42] Huo X Schnabel E Hughes K Frugoli J RNAi phenotypes and the localization of a protein:GUS fusion imply a role forMedicago truncatula PINgenes in nodulation. J 2006 25 156 165 10.1007/s00344-005-0106-y PMC2678931 19444321

[B43] Ehrhardt DW Atkinson EM Long SR Depolarization of alfalfa root hair membrane potential by Rhizobium meliloti nod factors Science 1992 256 998 1000 10.1126/science.10744524 10744524

