# Supplementary figures and images for: Simple and efficient methods to generate split roots and grafted plants useful for long-distance signaling studies in Medicago truncatula and other small plants
Source: Plant Methods. 2012 Sep 12;8:38. doi: 10.1186/1746-4811-8-38 (PMC3493353; doi:10.1186/1746-4811-8-38)

## Slide 1
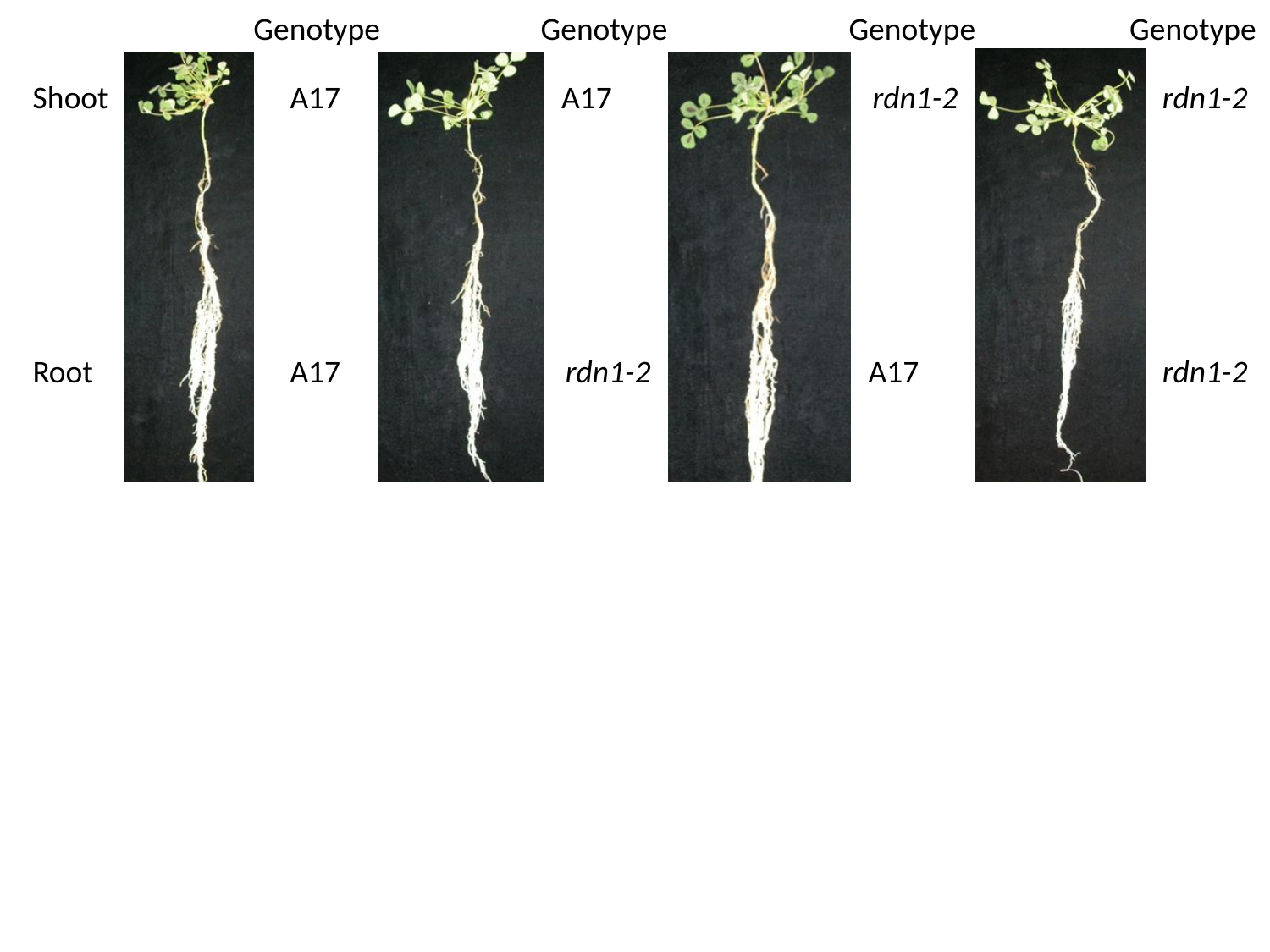

Genotype
Genotype
Genotype
Genotype
Shoot
A17
A17
rdn1-2
rdn1-2
Root
A17
rdn1-2
A17
rdn1-2

Supplement: Additional file 1 — Figure S1. Washed reciprocally grafted plants harvested from the soil ready for nodule counting showing large, healthy root systems. [file 1746-4811-8-38-S1.pptx]
